# Supplementary material for: The interactome of intact mitochondria by cross-linking mass spectrometry provides evidence for coexisting respiratory supercomplexes
Source: Mol Cell Proteomics. 2017 Dec 8;17(2):216–32. doi: 10.1074/mcp.RA117.000470 (PMC5795388; doi:10.1074/mcp.RA117.000470)
Supplement: Supplemental Data [file supp_RA117.000470_133922_0_supp_23978_fzffwf.zip › spectra_annotation/mito_DR_spectra_annotation/415-1-4-1-5-1.pdf]

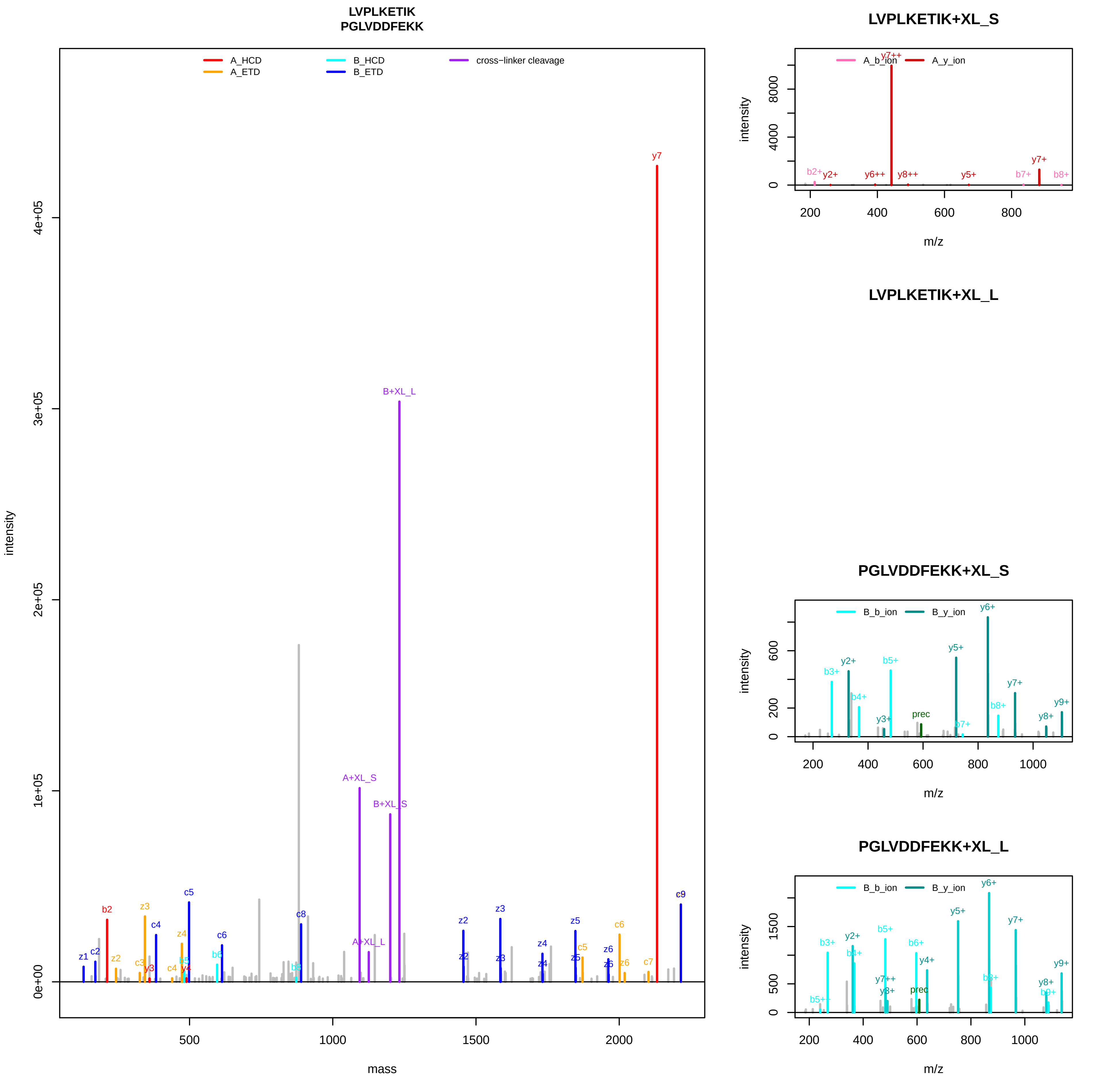

LVPLKETIK
PGLVDDFEKK
A_HCD
A_ETD
B_HCD
B_ETD
cross−linker cleavage
intensity
mass
0e+00
1e+05
2e+05
3e+05
4e+05
500
1000
1500
2000
y7
B+XL_L
A+XL_S
B+XL_S
A+XL_L
c9
LVPLKETIK+XL_S
A_b_ion
A_y_ion
y7++
y7+
b2+
y2+
y6++
y8++
y5+
b7+
b8+
m/z
LVPLKETIK+XL_L
PGLVDDFEKK+XL_S
B_b_ion
B_y_ion
y6+
y5+
y2+
b3+
b5+
b4+
y7+
y3+
prec
b7+
b8+
y8+
y9+
PGLVDDFEKK+XL_L
